# Fatty Acids Dysregulation Correlates with Lung Function in Idiopathic Pulmonary Fibrosis

**DOI:** 10.1007/s00408-025-00852-0

**Published:** 2025-10-14

**Authors:** Filippo Scialò, Raffaella Pagliaro, Monica Gelzo, Maria Gabriella Matera, Vito D’Agnano, Stefano Sanduzzi Zamparelli, Giuseppe Castaldo, Mario Cazzola, Andrea Bianco, Fabio Perrotta

**Affiliations:** 1 Department of Molecular Medicine and Medical Biotechnologies, University of Naples “Federico II”, Naples, Italy; 2 CEINGE-Biotecnologie Avanzate Franco Salvatore, Naples, Italy; 3 Department of Translational Medical Sciences, University of Campania ‘L. Vanvitelli’, Naples, Italy; 4 U.O.C. Clinica Pneumologica L. Vanvitelli, A.O. dei Colli, Monaldi Hospital, Naples, Italy; 5 Unit of Pharmacology, Department of Experimental Medicine, University of Campania ‘L. Vanvitelli’, Naples, Italy; 6 Division of Pneumology and Semi-Intensive Respiratory Therapy, A. Cardarelli Hospital, 80131 Naples, Italy; 7 Unit of Respiratory Medicine, Department of Experimental Medicine, University of Rome “Tor Vergata”, Rome, Italy

**Keywords:** Idiopathic pulmonary fibrosis, Fatty acids, Arachidonic acid, Oleic acid, Cis palmitoleic acid

## Abstract

**Background:**

Idiopathic pulmonary fibrosis (IPF) is a progressive lung disease with a poor survival rate and undefined molecular mechanisms. The identification of reliable biomarkers to help early diagnosis and predict disease progression is crucial for improving patient life. Although many biomarkers have been proposed, there is no consensus on reliable markers for IPF. Alterations in fatty acid (FA) metabolism have drawn increasing attention in the IPF pathogenesis.

**Methods:**

This single-center, prospective, cross-sectional study enrolled 35 IPF patients and 24 control participants. Demographic, clinical, and pulmonary function data were collected. FA profiles were compared between the two groups, with statistical analyses including chi-square tests, ANOVA, Spearman’s rank correlation, and ROC curve analysis.

**Results:**

We found significant differences in serum FA levels between IPF patients and controls. Cis-Palmitoleic acid (26.4 mg/L vs 22.1 mg/L; *p* = 0.04), oleic acid (457.6 mg/L vs 376.4 mg/L, *p* = 0.04), and elaidic acid (33.7 mg/dL vs 28.2 mg/L, *p* = 0.02) were increased in IPF patients, while arachidonic acid (79.7 mg/dL vs 97.9 mg/L, *p* = 0.01) levels were significantly lower compared to the control group. Spearman’s correlation analysis revealed positive correlations between these fatty acids. Notably, arachidonic acid levels showed a positive correlation with FEV1% (*r* = 0.348; *p* = 0.043) and FVC% (*r* = 0.431; *p* = 0.01), although ROC curve analysis indicated that this did not demonstrate strong diagnostic potential for IPF.

**Conclusion:**

In this study, we identified a dysregulation of cis-palmitoleic acid, oleic acid, elaidic acid, and arachidonic acid in IPF patients, indicating alterations in lipid metabolism and inflammatory pathways. Additionally, while arachidonic acid levels correlate with lung function, its diagnostic potential remains uncertain and warrants further evaluation in a larger patient population.

## Introduction

Idiopathic pulmonary fibrosis (IPF) is a chronic and progressive fibrotic lung disease with significant morbidity and poor survival outcomes [1]. Whilst progress has been made in the comprehension of IPF pathogenesis [2], the precise molecular pathways driving disease progression remain undefined. An early and accurate diagnosis, along with the ability to predict disease progression, could be life-changing for many patients, enabling effective disease management and timely personalized treatment. The clinical course varies widely among patients, making disease progression unpredictable. Specific biomarkers enabling IPF to be distinguished from other lung pathologies and provide insights into mechanisms of disease progression have already been proposed in literature. Examples include surfactant proteins A and D (SP-A and SP-D), which are elevated in both serum and bronchoalveolar lavage fluid (BAL) of IPF patients, reflecting alveolar epithelial damage—one of the hallmarks of IPF pathogenesis [3, 4]. Circulating levels of matrix metalloproteinases (MMP-8, MMP-9) and their tissue inhibitor TIMP-1 are also increased in serum [5], together with extracellular matrix remodeling biomarkers such as YKL-40, ICAM-1, LOXL2, and periostin [6–9]. In addition, expression of S100A4 has been identified as a fibrogenic feature in lung tissue from patients with IPF [10]. More recently, members of the sirtuin family (SIRT-1 and SIRT-3) have been reported to be elevated in serum of IPF patients [11]. KL-6, a glycoprotein secreted mainly by type II pneumocytes, is also increased in both serum and BAL of IPF patients, although it cannot be considered a specific biomarker since elevated levels have also been observed in several other lung diseases [12–14]. Among the dysregulated pathways in IPF, fatty acid (FA) metabolism is gathering increasing attention, as alterations in FA composition have been shown to promote pro-fibrotic traits in epithelial cells, fibroblasts, and myofibroblasts [15, 16]. However, while specific FA alterations have been identified in the serum of IPF patients, no consensus exists on the significance of changes for individual FAs [17–19]. Therefore, we have designed a prospective translational study to determine whether FA metabolism is altered in our IPF population and to explore the potential of specific FAs as IPF biomarkers.

## Material and Methods

### Study Population

This study was a single-center, prospective, cross-sectional investigation involving patients diagnosed with IPF. Participants were recruited from the Interstitial Lung and Rare Diseases Outpatient Clinic at the Vanvitelli Respiratory Diseases Clinic, Monaldi Hospital, Naples, Italy. Patients were included if they met the following criteria: (1) Confirmed IPF diagnosis based on the 2018 ATS/ERS/JRS/ALAT guidelines [20] within the past five years, (2) age ≥ 40 years, (3) ability to provide informed consent. Exclusion criteria included: (1) A history of significant exposure to environmental or occupational risk factors for pulmonary fibrosis (e.g., pro-fibrotic drugs, asbestos, beryllium, radiation, domestic birds), (2) a clinical diagnosis of any connective tissue disease (CTD), (3) a current diagnosis of asthma or chronic obstructive pulmonary disease (COPD), (4) an acute IPF exacerbation within the past three months. Demographic and anthropometric data—including sex, smoking history, and comorbidities were systematically recorded. All participants underwent pulmonary function testing, including spirometry, body plethysmography, to assess forced expiratory volume in 1 second (FEV1), forced vital capacity (FVC), total lung capacity (TLC), residual volume (RV), and single-breath diffusing capacity of the lungs for carbon monoxide (DLCO), following the 2022 ATS/ERS guidelines [21]. Tests were performed using the Vyntus BODY system (Vyaire Medical), and the following parameters were measured: FEV1, FEV1%, FVC, FVC%, FEV1/FVC, TLC, TLC%, RV, RV%, DLCO, and DLCO% [22, 23]. Additionally, arterial blood gas (ABG) analysis was conducted to assess pH, partial pressure of oxygen (pO_2_), partial pressure of carbon dioxide (pCO_2_), bicarbonates (HCO_3_^−^), and lactate levels. Functional capacity was evaluated using the 6-min walk test (6MWT), performed according to ATS guidelines [24]. The Gender-Age-Physiology (GAP) index was calculated to stratify IPF patients into three risk stages based on clinical (sex, age) and physiological (FVC, DLCO) parameters, providing an estimate of 1-, 2-, and 3-year mortality [25]. To enhance prognostic accuracy, the Distance-Oxygen-Gender-Age-Physiology (DO-GAP) index was also determined by incorporating 6MWT distance and exertional hypoxemia [26]. Control subjects, matched for age e and sex to the ILD population under study, and were consecutively recruited at the same centre among individuals without evidence of interstitial lung diseases. This study was conducted in compliance with the Declaration of Helsinki and received approval from the local Ethics Committee (reference number 7721). All participants provided written informed consent before enrolment.

### Statistical Analyses

Baseline characteristics of the population were compared between the control group and IPF patients. In particular, the X^2^ test or Spearman’s rank correlation (Rho) was applied to categorical variables according to their distribution, while the Wilcoxon-Mann–Whitney test was used for continuous variables to identify significant differences between the two groups (controls and IPF). For univariate correlations, Pearson’s or Spearman coefficient were used as appropriate. These statistical methods provide a comprehensive understanding of the clinical and biochemical differences between the two groups, as well as the relationships among the fatty acids studied. Finally, we evaluated the diagnostic potential of significant FAs for IPF using Receiver Operating Characteristic (ROC) curves. A *p*-value < 0.05 was considered statistically significant.

### Serum Fatty Acid Profile

Fatty acid profiles were analyzed as described in [27]. Briefly, serum were collected from IPF patients and controls at the time of pulmonary function tests (PFTs). Samples were then centrifuged at 3500 rpm form 15 min and stored at -80 °C. The chloroform extract solution, corresponding to 100 uL of patient serum was employed for the analysis of fatty acids after a transesterification step using BF3-methanol. Lipids were analyzed by gas chromatography and detected by flame ionization detector GC-FID (HP-5890, Agilent) controlled by a workstation equipped with MassLab 3.4 software [28].

## Results

### IPF Patients Show Dysregulated Serum Fatty Acid Levels

**Fig. 1:**
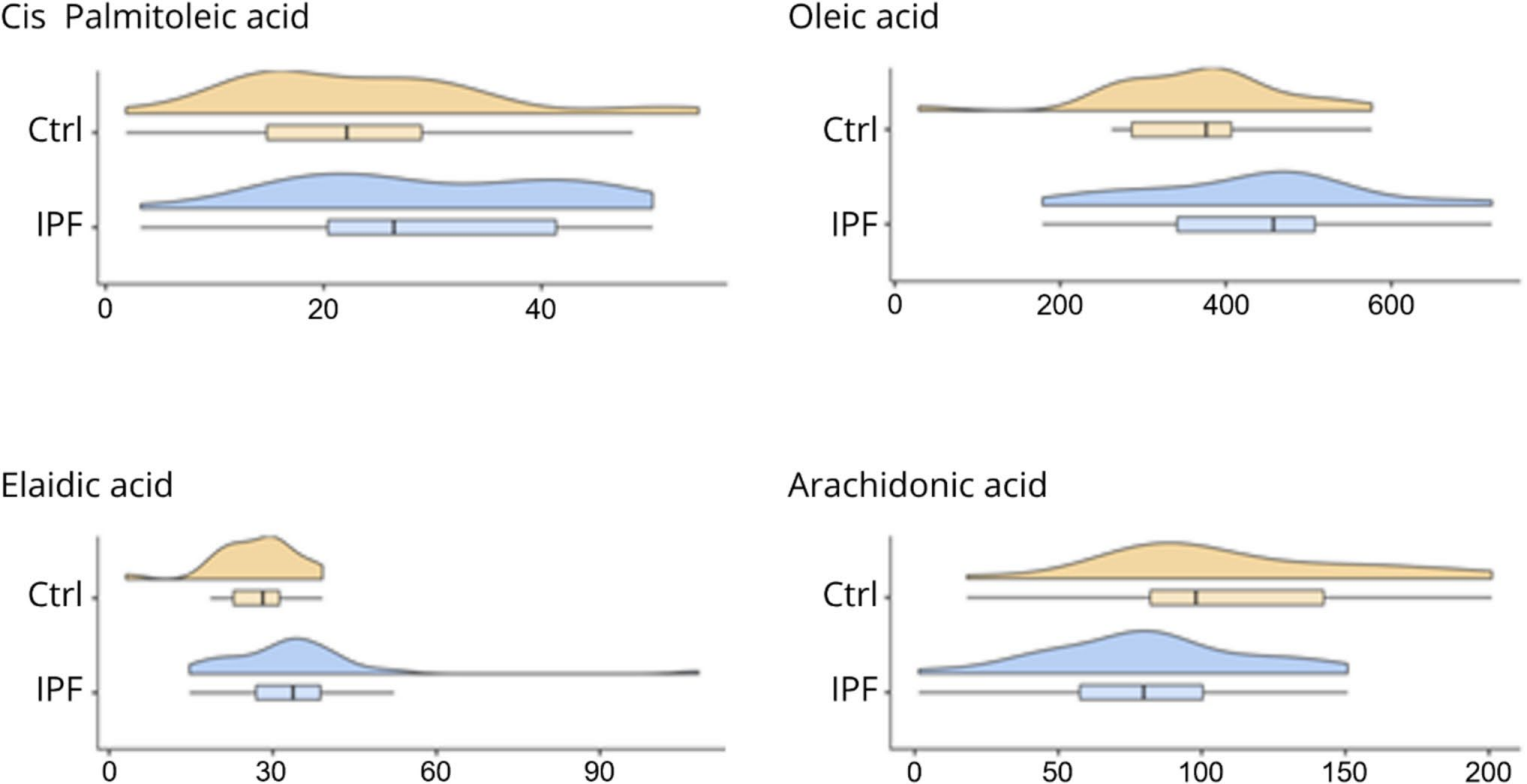
Comparison of fatty acid levels between the IPF and control groups

In this study, a total of 35 patients with a diagnosis of IPF and 24 control patients were enrolled. Among the IPF cohort, 30 were receiving antifibrotic treatment (21 with Nintedanib and 9 with Pirfenidone), whereas 5 patients were not on antifibrotic therapy. The baseline characteristics of patients included in our study are summarized in Table 1. Our analysis indicated that there were no significant differences between the IPF and control groups in terms of age, gender, BMI, smoking status, or the presence of comorbidities analyzed. Regarding smoking habit, 29 patients in the IPF group had a history of heavy smoking [29], and 2 patients were still current smokers at the time of enrolment. In the control group, 20 patients had a history of smoking, while 4 patients had no history of smoking (Table 1). Our analysis revealed that cis-palmitoleic acid (*p* = 0.04), oleic acid (*p* = 0.04), elaidic acid (*p* = 0.02), and arachidonic acid (*p* = 0.01) exhibited statistically significant differences between the two groups (Table 2). In particular, we observed higher levels of cis-palmitoleic acid (26.4 mg/L vs 22.1 mg/L), oleic acid (457.6 mg/L vs 376.4 mg/L), and elaidic acid (33.7 mg/L vs 28.2 mg/L) in the IPF group compared to the control group. Conversely, arachidonic acid levels were lower in the IPF group (79.7 mg/L vs 97.9 mg/L) compared to the control group (Table 2). There is a noticeable shift in the distribution patterns of the four FAs, with variations between the two populations. In particular, the average levels of cis-palmitoleic acid, oleic acid, and elaidic acid are increased in the IPF group, with some patients exhibiting abnormally high levels, especially for oleic and elaidic acids. In contrast, the mean average of arachidonic acid is decreased in the IPF population with some patients showing critically low levels (Fig. 1). Interestingly, the Spearman’s correlation analysis (Rho) (Table 3) resulted in a strong positive correlation between cis-palmitoleic acid and oleic acid (Rho = 0.606, *p* < 0.001), as well as between cis-Palmitoleic acid and elaidic acid (Rho = 0.620, *p* < 0.001). Additionally, a positive correlation was also found between cis-palmitoleic acid and arachidonic acid (Rho = 0.455, *p* = 0.007) and between oleic acid and elaidic acid (Rho = 0.681, *p* < 0.001).

**Table 1:** Baseline characteristics of our population divided into two groups (controls and IPF patients)

Characteristics	Controls (*N* = 24)	IPF (*N* = 35)	*P* value
Age, years	72.0 (68.0–75.6)	75.0 (68.1–77.1)	0.213
Male sex, n (%)	16 (67%)	27 (77%)	0.372
BMI, kg/m^2^	26.6 (24.6–28.5)	26.9 (24.9–29.8)	0.523
Smoking history, *n* (%)	Never 4 (17%)	Never 4 (11%)	0.442
	Former 20 (83%)	Former 29 (83%)	
	Current 0 (0%)	Current 2 (6%)	
Systemic hypertension, *n* (%)	14 (58%)	20 (57%)	0.932
Diabetes, *n* (%)	6 (25%)	9 (26%)	0.952
Dyslipidemia, *n* (%)	6 (25%)	13 (37%)	0.332
Coronary artery disease, n (%)	5 (21%)	6 (17%)	0.722
Stroke, n (%)	4 (17%)	3 (9%)	0.342
Chronic heart failure, n (%)	1 (4%)	3 (9%)	0.512
Atrial fibrillation, *n* (%)	1 (4%)	3 (9%)	0.512
Chronic kidney disease, n (%)	5 (21%)	4 (11%)	0.322
COPD, n (%)	1 (4%)	2 (6%)	0.792
GERD, n (%)	4 (17%)	12 (34%)	0.132
OSA, n (%)	4 (17%)	2 (6%)	0.172
Malignancy, *n* (%)	2 (8%)	5 (14%)	0.492

*BMI* body mass index, *COPD* chronic obstructive pulmonary disease, *GERD* gastroesophageal reflux disease, *OSA* obstructive sleep apnea

**Table 2:** Distribution of various fatty acids in two populations (controls Vs IPF patients)

Fatty acids	Controls (median [IQR])	IPF (median [IQR])	*P* value
Myristic acid (mg/L)	12.7 (9.9–17.7)	14.3 (10.6–19.5)	0.433
Trans Palmitoleic acid (mg/L)	5.5 (4.9–6.7)	6.7 (5.3–8.3)	0.143
Cis palmitoleic acid (mg/L)	22.1 (14.3–29.1)	26.4 (19.6–41.6)	0.043
Palmitic acid (mg/L)	413.7 (362.4–483.6)	444.1 (359.3–546.1)	0.253
Nonadecanoic acid (mg/L)	2.6 (2.3–3.2)	2.9 (2.7–3.2)	0.313
Margaric acid (mg/L)	6.1 (5.8–7.4)	6.8 (5.8–8.3)	0.333
Alpha-linolenic acid (mg/L)	4.2 (2.8–6.1)	3.5 (2.6–4.6)	0.163
Linolelaidic acid (mg/L)	380.2 (342.0–444.8)	352.1 (269.5–409.4)	0.283
Oleic acid (mg/L)	376.4 (285.5–406.9)	457.6 (337.8–508.3)	0.043
Elaidic acid (mg/L)	28.2 (22.5–31.3)	33.7 (26.2–38.8)	0.023
Stearic acid (mg/L)	178.2 (158.6–198.4)	178.8 (120.7–212.3)	0.963
Arachidonic acid (mg/L)	97.9 (81.4–142.9)	79.7 (57.1–101.4)	0.013
Dihomo-gamma-linolenic acid (mg/L)	37.5 (23.7–43.4)	34.4 (22.3–44.3)	0.673
Erucic acid (mg/L)	25.6 (8.2–29.4)	30.4 (8.4–33.7)	0.253
Cervonic acid (mg/L)	21.4 (19.8–32.4)	19.8 (13.4–24.1)	0.063
Colesta-3,5-diene (mg/L)	16.6 (8.2–37.7)	21.9 (13.3–43.4)	0.123

*IQR* interquartile range

**Table 3:** Correlations among fatty acids in IPF patients

		Cis Palmitoleic acid	Oleic acid	Elaidic acid	Arachidonic acid
Cis Palmitoleic acid	Spearman’s Rho	–			
	*p* value	–			
Oleic acid	Spearman’s Rho	0.606^***^	–		
	*p* value	< .001	–	–	
Elaidic acid	Spearman’s Rho	0.620^***^	0.681^***^	–	
	*p* value	< .001	< .001		
Arachidonic acid	Spearman’s Rho	0.455^**^	0.423^*^	0.389^*^	–
	*p* value	0.007	0.012	0.022	–

### Arachidonic Acid Serum Levels Correlate with FEV1% and FVC%

**Fig. 2:**
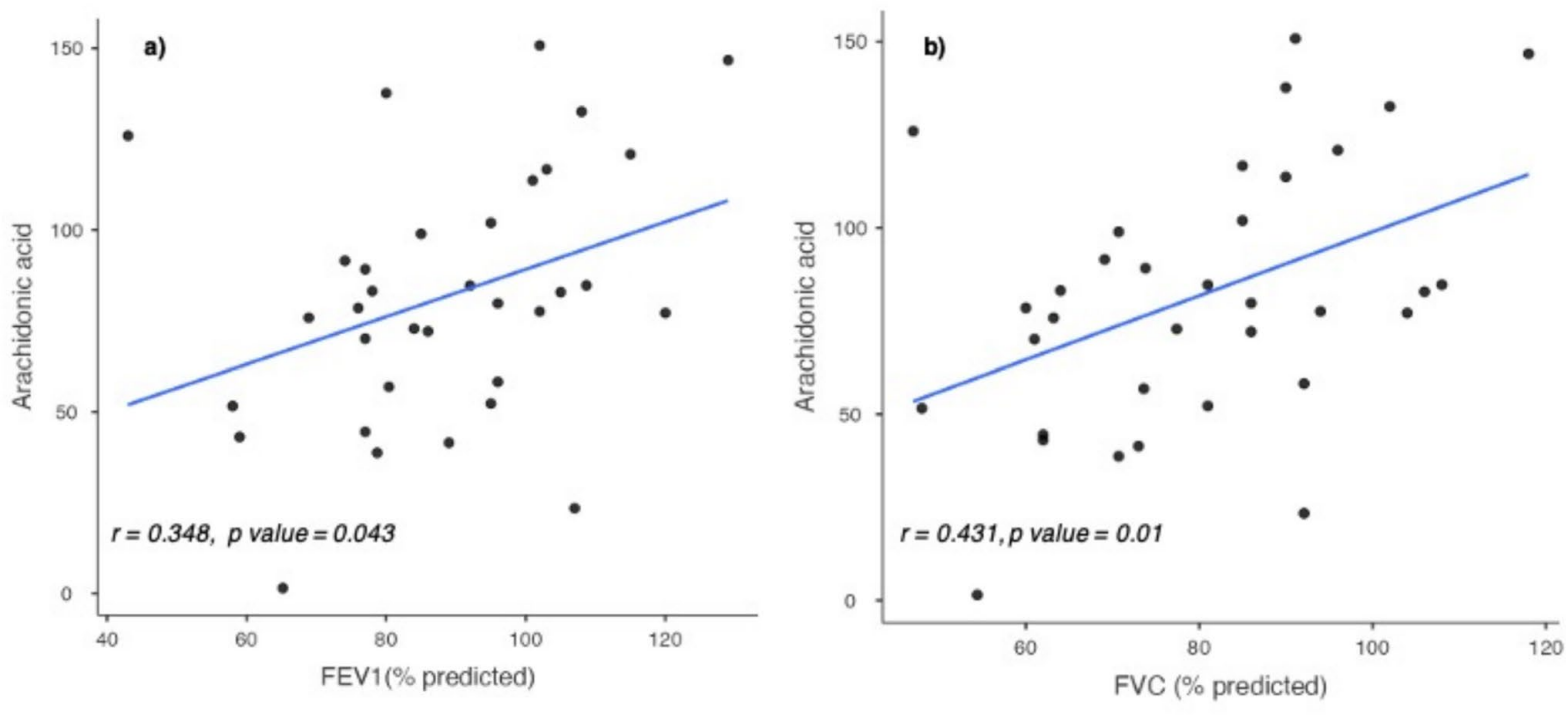
**a** and **b** Correlations between arachidonic acid levels and FEV1% and FVC%

**Fig. 3:**
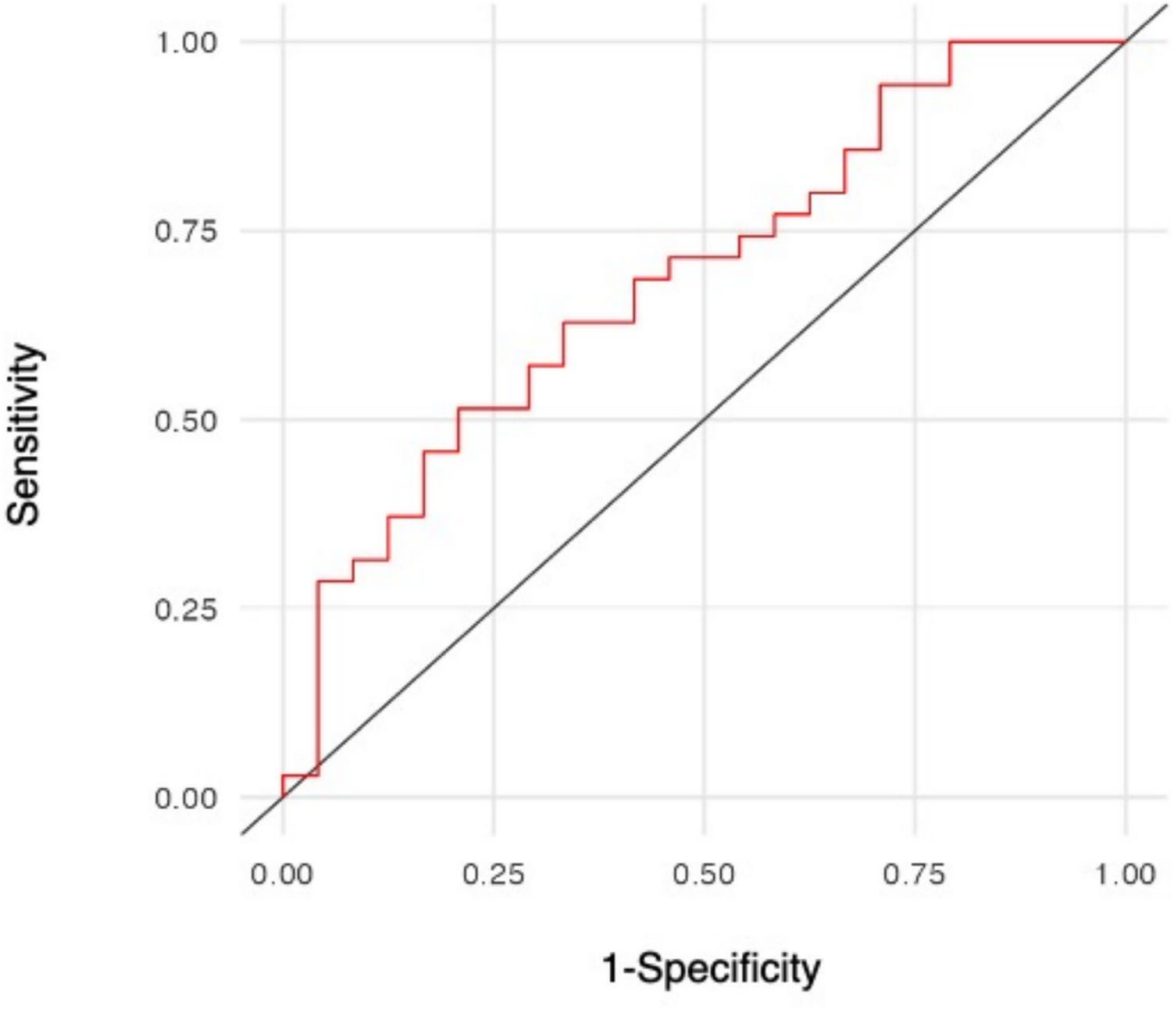
ROC curves to evaluate the diagnostic performance of arachidonic acid as a biomarker for IPF

We assessed whether a correlation existed between the four FAs and lung function tests. While we could not find any correlation (data not shown) for cis-palmitoleic, oleic and elaidic acid, arachidonic acid showed a statistically significant correlation (*p* = 0.04) with FEV1% (Fig. 2a). The CI for the correlation coefficient, calculated at a 95% confidence level, ranged from 0.011 to 0.614. The correlation coefficient (*r* = 0.348; *p* = 0.043) suggests a moderate positive relationship between arachidonic acid levels and FEV1%. Interestingly, the same analysis demonstrated that a positive correlation also existed between arachidonic acid and FVC%, with a confidence level of 95% ranging from 0.108 to 0.671. Also, in this case, the correlation coefficient (*r* = 0.431; *p* = 0.01) indicated a positive association between the two variables (Fig. 2b). No correlation was found between cis-palmitoleic, oleic and elaidic acid and FEV1% (data not shown). To further understand whether arachidonic acid could potentially be used as a biomarker for IPF, we analyzed its diagnostic performance using a ROC curve. This analysis indicated that although arachidonic acid appears to be linked with FEV1% and FVC%, statistical significance was not achieved as reflected by the low Youden’s Index (0.262) and moderate AUC (0.687) (Fig. 3).

## Discussion

In this study, a distinct dysregulation of FA metabolism in IPF has been identified. Indeed, IPF patients exhibited significantly increased serum levels of cis-palmitoleic acid, oleic acid, and elaidic acid, alongside decreased levels of arachidonic acid. These observed elevations are consistent with emerging literature suggesting that lipid dysregulation may contribute to the fibrotic process [19, 30–32]. In this respect, an aberrant de novo lipogenesis might be correlated with extracellular matrix invasion by fibroblasts in IPF [33, 34]. As demonstrated by Jung and colleagues, not only do FA synthase (FASN) levels directly correlate with the degree of fibrosis, but its inhibition reduces the expression of profibrotic genes and stabilizes or improves lung function in a bleomycin model of lung fibrosis [33]. Cis-Palmitoleic acid has been demonstrated to have a role in metabolic regulation and inflammation [35–37]. Elevated levels may reflect a compensatory or maladaptive response in the context of cellular stress and inflammation associated with IPF [38]. Oleic Acid is a monounsaturated fatty acid known to influence cell membrane fluidity and signalling [39]. Its increased serum levels might modulate the activity of inflammatory mediators and contribute to the remodelling of lung tissue, as suggested by previous studies [40, 41]. Elaidic Acid is typically less abundant in physiological conditions and is known for its pro-inflammatory properties. Its elevation in IPF patients could exacerbate inflammatory signalling, thereby promoting fibrotic changes [42–44]. Although data specifically linking elaidic acid to IPF are limited, the general pro-inflammatory role of trans fatty acids supports a potential contribution to disease pathology. Conversely, the reduced levels of arachidonic acid in IPF patients are particularly noteworthy [16, 45–48]. Arachidonic acid is a well-established precursor for eicosanoids, which are critical mediators of both pro-inflammatory and anti-inflammatory responses [48]. A decrease in circulating arachidonic acid may indicate its increased conversion to downstream mediators during active fibrotic or inflammatory processes. Recent studies have suggested that dysregulated eicosanoid metabolism, characterized by overproduction of LTs and deficiency of PGE2, promotes the development and progression of lung fibrosis [49–52]. In particular, an imbalance in the production of AA metabolites has been described in IPF patients with high levels of LTC4, LTB4 and PGE2 in lung tissue [53–55]. Whilst PGE2 is found to be decreased in bronchoalveolar lavage fluid (BALF) PGF2α levels are increased in the plasma of IPF patients. Interestingly, the elevated levels of PGF2α in the plasma are negatively correlated with lung function measurements in terms of FVC and DLCO [56–58]. The distinct dysregulation of serum fatty acid profiles observed in our IPF cohort aligns with an expanding body of evidence implicating lipid metabolism as a critical yet underexplored determinant of IPF pathogenesis. Mechanistic studies indicate that distinct fatty acids differentially modulate fibrogenesis, suggesting that IPF pathogenesis arises from an imbalance between harmful and protective lipid mediators. Unlike the profibrotic and proapoptotic effects of cis-palmitoleic, oleic, and elaidic acids observed in our study, stearic acid exerts antifibrotic effects by attenuating transforming growth factor-ß1 (TGF-β1)–mediated fibroblast activation and proliferation, thereby suggesting a potential protective role for specific fatty acids [51, 59]. Kim et al. recently reported that higher ω-3 fatty acid levels, including docosahexaenoic acid (DHA) and eicosapentaenoic acid (EPA), were associated with preserved DLCO% and improved transplant-free survival in IPF, underscoring their protective effects on alveolar-capillary function [60]. Corroborating our study, Faverio et al. found that reduced serum levels of arachidonic acid and DHA correlated with higher GAP stage and more rapid disease progression, alongside altered very-long-chain fatty patterns suggestive of peroxisomal dysfunction, including nervonic, lignoceric, and cerotic acid [40]. Arachidonic acid levels correlated positively with lung function, indicating that lower concentrations are associated with impaired pulmonary performance; however, modest ROC AUC values suggest limited diagnostic utility, supporting a role in mechanistic insight rather than as a stand-alone biomarker. Similarly, Roman et al. reported circulating lipid derivatives such as acylcarnitines, ceramides, and sphingomyelins as markers of disease severity measured as reduced DLCO% and adverse outcomes, predicting mortality independently of antifibrotic therapy [61]. These results complement our findings by suggesting that lipidomic signatures may serve as integrative biomarkers of functional decline and prognosis in IPF. Finally, evidence from ILD populations beyond IPF highlights the systemic relevance of ω-3 fatty acid status. Taken together, our findings and prior literature indicate that lipid dysmetabolism constitutes an active driver of IPF pathobiology, shaping fibrogenesis, lung function, prognosis, and radiographic manifestations. Moreover, the composition of circulating fatty acids not only reflects disease severity and outcomes but also underscores the potential of integrated metabolic profiling, in conjunction with lung function and imaging, as a strategy for risk stratification and therapeutic targeting in IPF. Given the relatively small sample size, these findings indicate that further studies with a larger cohort are necessary to determine whether arachidonic acid could serve as a robust standalone biomarker for IPF diagnosis. Interestingly, Spearman’s correlation analysis revealed strong positive correlations among cis-palmitoleic acid, oleic acid, and elaidic acid, hinting at the possibility of a coordinated regulation or shared metabolic pathway disruption in IPF. These interrelationships suggest that the dysregulation of one lipid species may influence or reflect alterations in others, thereby contributing collectively to the fibrotic milieu. Additional research in integrated lipid metabolic networks and pulmonary fibrosis is required in order for the mechanisms behind these correlations to be fully understood.

## Conclusions

Our study provides further evidence demonstrating that specific alterations in serum FAs levels are a hallmark of IPF. The increase in cis-palmitoleic, oleic, and elaidic acids, coupled with a reduction in arachidonic acid, not only reflects disturbed lipid metabolism but also appears to be linked to lung function impairment. These insights expand our understanding of the metabolic derangements underlying IPF and suggest that targeting lipid pathways might represent a novel therapeutic strategy. Further elucidation of these metabolic interactions is important for improving both diagnostic and treatment modalities in IPF. The limitations of our study include the relatively small sample size and the single-center design that may restrict the generalizability of our results. In addition, the cross-sectional nature of the study precludes any assessment of temporal changes or causality between FAs alterations and disease progression. We also recognise that, while we focused on four FAs of interest, other lipid species not analyzed here could also play significant roles in IPF pathogenesis

## Data Availability

All data generated or analyzed during this study are included in this article. Further inquiries can be directed to the corresponding authors.
